# Special Issue: Recent Research on Hospital-Acquired Bloodstream Infections

**DOI:** 10.3390/pathogens12070906

**Published:** 2023-07-04

**Authors:** Petros Ioannou, Diamantis P. Kofteridis

**Affiliations:** 1 School of Medicine, University of Crete, 71003 Heraklion, Greece; 2 Internal Medicine Department, University Hospital of Heraklion, 71110 Heraklion, Greece

Hospital-acquired infections (HAIs) are infections that occur in patients 48 h after admission to hospital. They represent a significant cause of morbidity, mortality, and increased hospital costs [1,2,3]. In the last few decades, these infections have been the focus of clinicians, infection control nurses, and administrative agencies in attempts to reduce their incidence [4]. Point prevalence surveys in different hospitals and settings have demonstrated HAI rates between 5% and 12% [5,6,7,8,9], with bloodstream infections (BSIs) being the most common infections among HAIs [5,6,7,8,9]. BSIs can lead to death or significant complications if left untreated, such as endocarditis, spondylodiscitis, and meningitis [10].

Hospital-acquired BSIs (HABSIs) can possess a mortality rate of up to 20%, even though this largely depends on the origin of the infection [10,11]. The microbiology of such infections most commonly involves *Staphylococcus aureus* and very commonly methicillin-resistant *S. aureus* (MRSA), as well as *Enterococcus*, including vancomycin-resistant *Enterococcus* (VRE) and Gram-negative bacteria such as *Escherichia coli*, *Klebsiella pneumoniae*, *Pseudomonas aeruginosa*, and *Acinetobacter baumannii*, [5,12]. Antimicrobial resistance complicates the treatment of patients with HABSIs, since pathogens that are multidrug-resistant (MDR), extensively-drug-resistant (XDR), and even pan-drug-resistant (PDR) are increasingly prevalent and are associated with high mortality rates [13,14].

Studies providing data on HABSIs, such as point prevalence surveys and epidemiological studies are of increasing importance as they provide data that help to increase our understanding of the problems caused by these significant infections. Understanding the magnitude of the problem is the first step toward its management. Infection control practices and even antimicrobial stewardship interventions may lead to a reduction in the incidence of HABSIs and a reduction in their antimicrobial resistance, thus reducing mortality and associated hospital costs [15,16]. Furthermore, studies on the microbiology of these infections are of practical value since they provide important information on their etiology and patterns of antimicrobial resistance. These studies can help clinicians choose appropriate empirical treatment before the results of cultures are available [17].

This Special Issue aims to bring together original studies as well as comprehensive narrative or systematic reviews related to epidemiology, microbiology, clinical characteristics, treatment, and outcomes of patients with HABSIs. Moreover, studies emphasizing infection control and antimicrobial stewardship of these infections are additionally welcome.
